# Œsophageal Obstruction.—I

**Published:** 1908-08-01

**Authors:** 


### SURGERY.

### ŒSOPHAGEAL OBSTRUCTION.—I.

The recognition of the presence of œsophageal obstruction is rarely, if ever, a matter of any difficulty, because the signs and symptoms presented are very characteristic. The patient experiences gradually increasing difficulty in the ingestion of solid food accompanied by pain in the neck on attempting to do so. After a time the passage of solid food, however well masticated, becomes quite impossible; and if a solid diet is attempted it is regurgitated almost immediately. Instantaneous vomiting after the ingestion of food is almost pathognomonic of œsophageal obstruction; if the stomach is at fault, there is an interval of at least half an hour before the vomiting begins.

If the patient does not at this period consult a medical man, his own experience induces him to modify his diet, and thus it happens that he comes to subsist entirely on milk, and finally even fluids are unable to pass. Whatever be the cause of the obstruction, emaciation necessarily ensues, and the patient loses flesh because he is not getting sufficient nutrition, and therefore, although malignant disease is the commonest cause of œsophageal obstruction, it is not justifiable to argue that because a patient has œsophageal obstruction and is obviously much wasted, the case is necessarily one of carcinoma.

There are many causes of this condition, and they may be classified according to whether the obstruction is due to some abnormality (*a*) in the lumen of the tube, (*b*) in the wall, or (*c*) which presses on the œsophagus from without. In the first category may be mentioned foreign bodies and polypi, but it must be confessed that obstruction arising from either of them is excessively rare; in the second, malignant disease, or cicatrisation of an ulcer caused by irritant bodies or by syphilis; in the third, an aneurysm of the transverse arch of the aorta is the commonest, and must always be borne in mind; but at the same time other conditions occasionally produce the same effect, *e.g.* enlarged bronchial or mediastinal glands, thyroid tumours, abscesses secondary to spinal caries, and empyemata. Lastly, an obstruction may be due to no organic cause at all, but be entirely functional in origin.

Thus it will be seen that there are a large number of possible causes of œsophageal obstruction which have to be kept in mind in arriving at a diagnosis, although the great majority of all cases are caused by carcinoma; and it follows that a thorough examination of the patient is essential.

The history is of value, since if the patient is known to have swallowed a foreign body or accidentally taken a strong irritant, such as sulphuric acid, the cause is obvious. Examination of the neck will enable one to exclude obstruction due to the pressure of a thyroid tumour, while the discovery of enlarged hard glands in the posterior triangle on the left side is highly suggestive of malignant disease. Carcinoma of the œsophagus and of the stomach is very prone to cause enlargement of the glands at the base of the neck on the left side. In his "Practice of Medicine" Osler writes: "The cervical lymph glands are frequently enlarged, and may give an early indication of the nature of the trouble."

The throat and fauces should next be examined, and then an œsophageal bougie should be passed; but before doing this the possibility of the obstruction being due to an aneurysm of the transverse arch of the aorta should be rigidly excluded. The signs to look for are dulness over the sternum with or without an aortic regurgitant murmur; tracheal tugging; change in the voice, due to pressure on the left recurrent laryngeal nerve; and a difference in the radial pulses, that on the left side being diminished and delayed. If any of these are present, an œsophageal bougie should on no account be passed, because there is a definite risk of rupturing the aneurysm.

If, however, there is no evidence of aneurysm, the examination may be proceeded with. Whenever it is possible, it is preferable to give an anæsthetic. Not only is the passage of a bougie a very unpleasant business for the patient, but obstruction, due to functional causes, may be detected in this way, since if the bougie passes easily into the stomach, it is obvious that there can be no organic obstruction. In order to know when the point of the bougie has passed into the stomach, it must be remembered that the cardiac orifice lies at a distance of sixteen inches from the incisor teeth, and the length which has been inserted can be measured on its withdrawal. The passage of the bougie is a simple manipulation; it is best done by passing the two first fingers of the left hand back to the fauces and guiding the point of the bougie between them with the right until it impinges on the pharyngeal wall, when slight pressure will cause it to pass down the pharynx.

If the obstruction be due to malignant disease, the progress of the bougie will be arrested at one of three points, (*a*) opposite the cricoid cartilage, (*b*) where the left bronchus crosses the œsophagus, or (*c*) at the cardiac orifice of the stomach, since these are the usual sites of the disease. These are the narrowest points of the œsophagus, and the fact is held to uphold irritation as a predisposing factor.

If there be a carcinoma, a small amount of offensive blood-stained material may be found on the point of the bougie. This should be collected and submitted to microscopical examination if there is sufficient of it; and in this way the diagnosis can, of course, be clinched.

It has been said that the involvement of the left recurrent laryngeal nerve is suggestive of thoracic aneurysm, but it must be remembered that in the later stages an œsophageal carcinoma may involve and press upon one or both recurrent laryngeal nerves, leading to more or less complete abductor paralysis and change of voice. The condition of the cords should therefore be examined with the laryngoscope.

The form of disease generally found is a squamous-celled carcinoma, as might be expected; but occasionally the columnar-celled type is met with. This is explained on the ground that during fœtal life the œsophagus is lined with columnar epithelium.

